# The first complete chloroplast genome of *Briggsia chienii* W. Y. Chun and its phylogenetic position within Gesneriaceae

**DOI:** 10.1080/23802359.2022.2087551

**Published:** 2022-06-24

**Authors:** Xiaolan Xu, Mengmeng Shi, Linchun Shi, Wujun Zhang, Jinxin Liu, Xinle Duan

**Affiliations:** a College of Animal Science (College of Bee Science), Fujian Agriculture and Forestry University, Fuzhou, China; b Hebei Key Laboratory of Study and Exploitation of Chinese Medicine, Chengde Medical University, Chengde, China; c Institute of Medicinal Plant Development, Chinese Academy of Medical Sciences, Peking Union Medical College, Beijing, China; d Institute of Agricultural Bioresource, Fujian Academy of Agricultural Sciences, Research Center for Medicinal Plant, Fujian Academy of Agricultural Sciences, Fuzhou, Fujian, China

**Keywords:** *Briggsia chienii*, Chloroplast genome, phylogeny, genome structure, Gesneriaceae

## Abstract

*Briggsia chienii* W. Y. Chun 1946 is an endemic herbaceous perennial species distributed in southern China. In this study, we firstly characterized the complete chloroplast genome sequence of *B. chienii* and provided new molecular resources for promoting its conservation and taxonomic assignment. Its complete chloroplast genome is 154,082 bp in length and contains the typical quadripartite structure of angiosperm plastome, including two inverted repeat (IR) regions of 25,447 bp, a large single-copy (LSC) region of 85,035 bp, and a small single-copy (SSC) region of 18,153 bp. The plastome contains 114 genes, consisting of 80 protein-coding genes, 30 tRNA gene, and 4 rRNA genes. The overall GC content in the plastome of *B. chienii* is 37.4%, which is lower than lots of angiosperm plastome. The phylogenetic result indicated that *B. chienii* exhibited the closest relationship with *Oreocharis cotinifolia* W. T. Wang 1983, and provided new information for the phylogeny relationship of genus *Briggsia.*

*Briggsia chienii* W. Y. Chun 1946 is an herbaceous perennial plant belonging to family Gesneriaceae. It is a Chinese endemic plant, and only can be found on moist rocks and grasses between 500 and 1000 meters of southwestern Zhejiang, southern Anhui, and eastern Jiangxi. The plants of *B. chienii* is stemless, all leaves are basal and stalked, and corolla is purple-red or purple with inside purple spots. *B chienii* is a medicinal plant that can also be utilized as a garden or indoor potted plant. The genus *Briggsia* was established in 1919 by Craib (Craib 1919), and have been considered to consist of 22 species and three varieties in the world, one of which is distributed in Sikkim, and the other three are shared by China, Myanmar, Bhutan, India, and Vietnam, and the remaining 18 species are endemic to China (Wang et al. 1998). According to previous studies, this genus should not be recognized as a valid group, and all of its species should be moved to other genera, including Glabrella, Loxostigma, and Oreocharis. The synonym of *B. chienii* should be *Oreocharis chienii* (Möller et al. 2014). However, there are limited researches on the genus *Briggsia*, and still insufficient morphological and molecular evidences to transfer all its member species to other gnus, especially for species *B. chienii*. Here, we reported and characterized the first complete chloroplast genome of *B. chienii* based on high throughput sequencing technology, and reconstructed the phylogeny relationships by utilizing the published chloroplast genome sequences of Gesneriaceae.

The original plants of *B. chienii* were collected from Fuxi village, Tangkou Town, Huangshan City in Anhui Province (N30°07′, E118°12′). And the plants were collected and identified by Xinlei Zhao (zhaoxinlei2009@sina.com, this sample was neither collected from a protected area nor listed on any endangered species list, such as CITES. And, under current law, what is not prohibited is what is allowed to be collected). These materials are deposited in the Institute of Medicinal Plant Development herbarium (herbarium code ‘IMD,’ NYBG: https://www.nybg.org/, Linchun Shi: lcshi@163.com) with the voucher number as HPAA0003. Genomic DNA was extracted from the leaf tissues using the plant genomic DNA extraction kit [Tiangen Biochemical Technology (Beijing) Co., Ltd, China] according to the protocol provided by the manufacturer with some modifications (Zhang et al. 2021). The quantity and quality of the total DNA were examined using Qubit 4.0 (Thermo Fisher Scientific Inc., USA). The sequencing library was constructed according to the TruSeq DNA PCR-free library preparation guide. The Illumina NovaSeq platform was employed to conduct the high-throughput sequencing, and approximately 1.8 Gb of clean data was generated with 150 bp paired-end read lengths. Trimmonmatic v0.38 was employed to remove the adapters and low-quality reads with the parameters ‘TruSeq3-PE.fa:2:30:10 LEADING:3 TRAILING:3 SLIDINGWINDOW:4:15 MINLEN:36’ (Bolger et al. 2014). The remaining reads were assembled into the complete chloroplast genome by using the organelle assembler GetOrganelle v1.7.3.5 with the parameters ‘-R 15 -k 21,45,65,85,105 -F embplant_pt’ (Jin et al. 2020), and validated by reads mapping using bowtie2 (Langmead and Salzberg 2012). The protein-coding, rRNA, and tRNA genes of *B. chienii* chloroplast genome were annotated by using the CPGAVAS2 online webserver (www.herbalgenomics.org/cpgavas2) (Shi et al. 2019). The complete chloroplast genome sequence of *B. chienii* was submitted to GenBank (Accession number: MZ868555).

The length of the *B. chienii* complete chloroplast genome sequence was 154,082 bp, and the total GC content was 37.4%. The genome displayed a typical quadripartite structure, including large single-copy (LSC) region (85,035 bp), small single-copy (SSC) region (18,153 bp) and a pair of inverted repeats IR regions (25,447 bp). There are 114 unique genes in the whole chloroplast genome sequence of *B. chienii*, including 80 protein-coding genes, 30 represented tRNA genes, and four denoted rRNA genes (*rrn23S*, *rrn16S*, *rrn5S*, and *rrn4.5S*). Among them, 19 genes were annotated as containing introns. Nine protein-coding genes and seven tRNA genes (*trnA-UGC*, *trnG-UCC*, *trnI-GAU*, *trnK-UUU*, *trnL-UAA*, *trnR-UCU*, *trnV-UAC*) contained one intron, and three protein-coding genes (*clpP*, *ycf3*, and *rps12*) contained two introns. In addition, small exons were annotated in *petB*, *petD*, and *rpl16* genes, and the length of their small exons were 6 bp, 8 bp and 9 bp, respectively. Moreover, *rps12* was identified as a trans-splicing gene.

To confirm the phylogenetic position of *B. chienii* in Gesneriaceae, a total of 18 complete chloroplast genomes were used for phylogenetic analysis based on the Maximum Likelihood (ML) method using RAxML v8.2.12 with 1000 bootstrap replicates (Stamatakis 2014). *Salvia mekongensis* E. Peter 1936 and *S. umbratica* Hance 1884 were used as the out groups. The phylogenetic result indicated that *B. chienii* exhibited the closest relationship with *Oreocharis cotinifolia* (Figure 1), and was consistent with the prior taxonomic suggestion (Möller et al. 2014). This study provided a useful molecular resource for its conservation and the phylogenetic studies of genus *Briggsia*.

**Figure 1.**
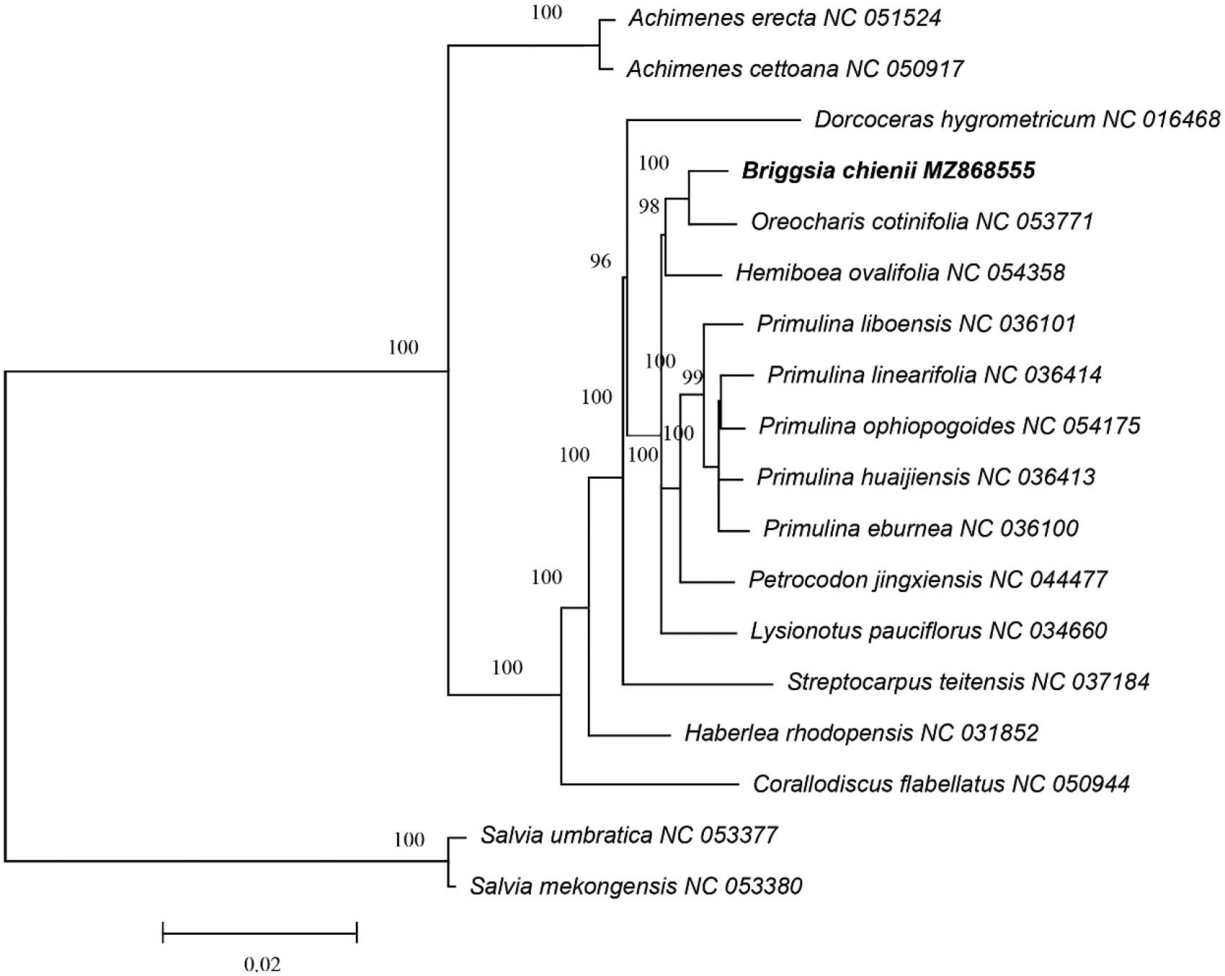
The phylogram tree recovered from 18 complete chloroplast genome sequences by RAxML. Their accession numbers can be found after the species names.

## Author contributions

X.X., J.L. and X.D. conceived and designed the experiments; M.S and L.S. performed the experiments; M.S and C.S. analyzed the data; M.S., L.S. and W.Z. contributed reagents/materials/analysis tools; X.X., J.L. and X.D. wrote the paper.

## Data Availability

The genom esequence data that support the findings of this study are openly available in GenBank of NCBI at (https://www.ncbi.nlm.nih.gov/) under the accession no. MZ868555. The associated BioProject, SRA, and Bio-Sample numbers are PRJNA682118, SRR16930608, and SAMN23005362, respectively.
